# Postmarketing commitments for novel drugs and biologics approved by the US Food and Drug Administration: a cross-sectional analysis

**DOI:** 10.1186/s12916-019-1344-3

**Published:** 2019-06-17

**Authors:** Joshua D. Wallach, Anita T. Luxkaranayagam, Sanket S. Dhruva, Jennifer E. Miller, Joseph S. Ross

**Affiliations:** 1 Department of Environmental Health Sciences, Yale School of Public Health, 60 College Street, 4th Floor, Room 411, New Haven, CT 06510 USA; 2 Collaboration for Research Integrity and Transparency (CRIT), Yale Law School, 157 Church Street, 17th Floor, Suite 1, New Haven, CT 06510 USA; 3 Center for Outcomes Research and Evaluation (CORE), Yale-New Haven Hospital, 1 Church Street, Suite 200, New Haven, CT 06510 USA; 4 University of Connecticut, Storrs, CT 06269 USA; 5 Department of Medicine, University of California, San Francisco School of Medicine, San Francisco, 94121 CA USA; 6 Section of Cardiology, San Francisco Veterans Affairs Health Care System, 4150 Clement St, Building 203, 2nd Floor Cardiology, San Francisco, CA 94121 USA; 7 Section of General Medicine, Department of Internal Medicine, Yale School of Medicine, P.O. Box 208093, New Haven, CT 06520-8093 USA; 8 National Clinician Scholars Program, Department of Internal Medicine, Yale School of Medicine, New Haven, 06510 CT USA; 9 Department of Health Policy and Management, Yale School of Public Health, 60 College Street, New Haven, CT 06510 USA

**Keywords:** Postmarketing commitments, Postmarketing requirements, FDA, Lifecycle evaluation, Pharmaceutical regulation

## Abstract

**Background:**

Postmarketing commitments are clinical studies that pharmaceutical companies agree to conduct at the time of FDA approval, but which are not required by statute or regulation. As FDA increasingly adopts a lifecycle evaluation process, greater emphasis will be placed on postmarket evidence as a component of therapeutic evaluation. Therefore, the objectives of this study were to determine how often postmarketing commitments agreed upon by pharmaceutical companies at first FDA approval lead to new clinical trials and to establish the characteristics and rates of completion and dissemination of postmarketing commitments.

**Methods:**

For new drugs and biologics approved in 2009–2012, we used public FDA documents, ClinicalTrials.gov, and Scopus, to determine postmarketing commitments and their characteristics known at the time of FDA approval; number subject to reporting requirements, for which FDA is required to make study status information available to the public (“506B studies”), and their statuses; and rates of registration and results reporting on ClinicalTrials.gov and publication in peer-reviewed journals for all clinical trials.

**Results:**

Among 110 novel drugs and biologics approved by the FDA between 2009 and 2012, 61 (55.5%) had at least one postmarketing commitment at the time of first approval. Of 331 total postmarketing commitments, 33 (10.0%) were for new clinical trials; 27 of these were 506B studies subject to public reporting requirements, of which 12 (44.4%) did not have a recent (i.e., up-to-date) or closed (i.e., fulfilled or released) status provided publicly by the FDA. Although two postmarketing commitments were insufficiently described in FDA records to perform searches on ClinicalTrials.gov, nearly all (28, 90.3%) of the 31 remaining postmarketing commitments for new clinical trials were registered on ClinicalTrials.gov. Among the registered trials, 23 (23 of 28, 82.1%) were classified as completed or terminated, of which 22 (95.7%) had reported results. When considering all 29 completed or terminated clinical trials, registered or unregistered on ClinicalTrials.gov, only half (14, 48.3%) were published in peer-reviewed journals.

**Conclusions:**

While only 15% of postmarketing commitments agreed to by pharmaceutical companies at the time of FDA approval were for new clinical trials, these trials were nearly always registered with reported results on ClinicalTrials.gov. However, only half were published, and despite FDA public reporting requirements, recent status information was often unavailable for 506B studies.

**Electronic supplementary material:**

The online version of this article (10.1186/s12916-019-1344-3) contains supplementary material, which is available to authorized users.

## Background

Under the US Food and Drug Administration’s (FDA) lifecycle evaluation process, it is assumed that the benefit-risk balance of drugs and biologics will continue to be monitored after approval [1, 2]. Although FDA currently has four authorities that can be used to require New Drug Application (NDA) sponsors (generally pharmaceutical companies) to conduct studies in the postmarket setting (i.e., “postmarketing requirements,” Additional file 1: Box S1, [3, 4]), additional clinical evidence that may not be generated through specific postmarketing requirements, including long-term drug effectiveness, duration of response, or efficacy in subgroup data, can be generated through “postmarketing commitments,” which are “studies or clinical trials that a sponsor has agreed to conduct, but that are not required by a statute or regulation” [4].

Prior to 2008, the term “postmarketing commitment” referred to all required, agreed-upon, or voluntary studies conducted by pharmaceutical companies after FDA drug approval (Additional file 1: Box S1) [4]. Although FDA had the authority to require certain studies after approval, approximately 90% of all postmarketing studies between 1990 and 2004 were agreed-upon commitments [5]. However, once the FDA Amendments Act (FDAAA) went into effect on March 25, 2008, FDA began to distinguish between legally required studies and clinical trials (i.e., postmarketing requirements) and those that pharmaceutical companies agree to conduct but are not required (i.e., postmarketing commitments). While postmarketing commitments are not formally mandated by FDA, certain postmarketing commitments are subject to reporting requirements. In particular, the Food and Drug Administration Modernization Act of 1997, which added section 506B (“506B studies”) to the Federal Food, Drug, and Cosmetic Act, requires drug manufacturers to provide the FDA with annual reports on the status of “clinical safety, clinical efficacy, clinical pharmacology, or nonclinical toxicology” postmarketing commitment studies [6]. Furthermore, FDA must publicly report on the status of these 506B postmarketing commitment studies [6]. However, according to 21 Code of Federal Regulations (CFR) 314.81(b)(2)(vii), and CFR 601.70, manufacturers of drugs, but not biologics, are required to report to the FDA for voluntary, chemistry, manufacturing, and controls, and product stability non-506B studies [3, 6] (Additional file 1: Box S2).

**Fig. 1:**
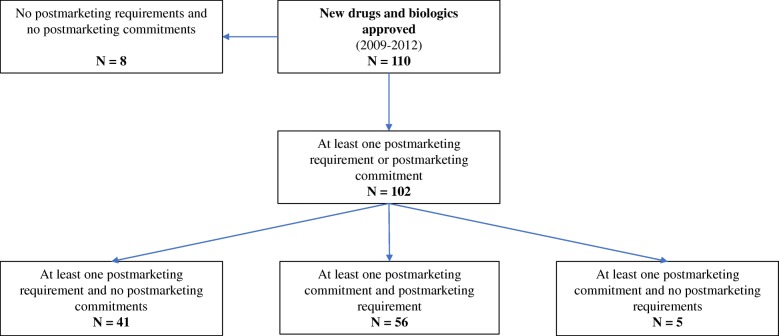
Postmarketing requirements and postmarketing commitments for 110 new drugs and biologics approved by the US Food and Drug Administration between 2009 and 2012

Prior studies have focused exclusively on the characteristics [7], completion [8, 9], and dissemination of postmarketing requirements [7, 10, 11]. For instance, among the 110 new drugs and biologics approved by FDA between 2009 and 2012, 97 (88.2%) had at least one postmarketing requirement at the time of approval (Fig. 1) [7]. Of the 437 total postmarketing requirements, 110 were for new clinical trials, which were often briefly described and were not consistently registered on ClinicalTrials.gov with reported results [7]. According to FDA, an average of 58 postmarketing commitments have been agreed upon per year since 2011 [12]. While this is significantly lower than the 264 postmarketing requirements per year, postmarketing commitments consistently made up approximately 19% of all commitments and requirements in 2015–2017 [12]. Therefore, postmarketing commitments may be a potentially important source of information about drug and biologic safety and effectiveness after market approval. In this study, we characterized the postmarketing commitments for all novel drugs and biologics approved between 2009 and 2012 using publicly available data sources, including their status and study characteristics, and for clinical trial postmarketing commitments, the rates and timeliness of registration and results reporting on ClinicalTrials.gov, and publication in peer-reviewed journals.

## Methods

### Study design and sample

As in prior work focused on postmarketing requirements [7], we used the publicly available Drugs@FDA database to identify and categorize all novel drug and biologic license applications (excluding generic drugs, reformulations, and combination therapies of non-novel therapeutic agents) first approved between January 1, 2009, and December 31, 2012 [13, 14]. As previously described [13], we characterized each new drug and biologic by date of approval, as a pharmacologic entity (small molecule) or biologic, and by orphan status. We then determined the first-approved indication for each new drug and biologic and whether applications were designated by FDA for priority review status [15]. The World Health Organization’s anatomic therapeutic classification system was used to categorize and group each indication into one of eight therapeutic areas [16].

### Identifying postmarketing commitments and postmarketing commitment features

One author (ATL) identified all postmarketing commitments and dates that the FDA sets for important milestones (i.e., final protocol submission, trial completion, and final report submission), which are outlined in the approval letters hyperlinked in the Drugs@FDA database (June 2018). These letters include a brief description of the study type and outline whether commitments are subject to certain reporting requirements (Additional file 1: Box S2). We then classified each postmarketing commitment into one of four study categories about the type of study required (Additional file 1: Box S3), calculated the length of each postmarketing commitment study description (word count), and abstracted key study design characteristics using a previously described approach [7].

### Status of postmarket studies

We used Postmarketing Study and Clinical Trial Requirements and Commitments Database Files to determine the status (i.e., *Pending*, *Ongoing*, *Delayed*, *Terminated*, *Submitted*, *Fulfilled*, or *Released*. Additional file 1: Box S4) of commitments specifically classified as subject to reporting requirements under 506B [17]. We downloaded the most recent Postmarketing Study and Clinical Trial Requirements and Commitments Database File on August 2, 2018 (representing data updated by FDA as of July 24, 2018). However, postmarketing commitments with *Fulfilled* and *Released* statuses are only displayed on the FDA’s online database for 1 year after the date of fulfillment or release. Therefore, previous Postmarketing Study and Clinical Trial Requirements and Commitments Database Files were located using the FDA.gov Archive. Studies were classified as not having a recent (i.e., up-to-date) or closed (i.e., *Fulfilled* or *Released*) status if we were unable to locate a database file outlining that they had been *Released* or *Fulfilled*. For these studies, we recorded (1) the most recent status that we could determine from FDA’s available archived files (e.g., *Delayed*) and (2) the date of the archived databases for each postmarketing commitment (e.g., October 31, 2010), as previously described [7]. We then performed additional Google searches using the terms “postmarketing requirement,” “postmarketing commitment,” “PMR,” or “PMC” in combination with the pharmaceutical company or drug brand name to determine whether pharmaceutical companies were publicly sharing their own information about postmarketing commitments (e.g., “Pfizer PMC” or “Pfizer postmarketing commitment”). For each Google search, we screened the first 100 results. Lastly, we reviewed all supplemental letters on the Drugs@FDA database to determine whether they included information regarding the fulfillment of postmarketing commitments. Abstractions were performed by one reviewer (ATL) in June and July 2018, and consistency and accuracy were verified by a second reviewer (JDW).

### Trial registration and results reporting on ClinicalTrials.gov and peer-reviewed publication

For all new clinical trials and all commitments that call for the completion and submission of the results from “ongoing” clinical trials (Additional file 1: Box S3), we determined study registration and results reporting on ClinicalTrials.gov (June and July 2018), as previously described [7]. If identified, for each registered clinical trial, one reviewer (JDW) abstracted study characteristics from the ClinicalTrials.gov registration. The primary outcome providing the highest level of evidence was recorded. For instance, for trials with multiple primary efficacy outcomes, we considered clinical outcomes the highest level, followed by clinical scales and surrogate markers [18]. A third reviewer (SSD) repeated all searches for trials that were determined to be unregistered, and uncertainties were discussed with the senior investigator (JSR).

For all clinical trials with a *Completed* or *Terminated* status on ClinicalTrials.gov for which results reporting would be expected, we abstracted whether any study results were reported and/or corresponding articles were published. For *Completed* or *Terminated* trials without reported results, we determined whether the date of final data collection for the prespecified primary outcome measure(s) (primary completion date) was within 12 months of the follow-up date (July 2018). According to the Final Rule for Clinical Trials Registration and Results Information Submission (“Final Rule”), submission of final results information is required “not later than 1 year after the completion date” [19]. For all clinical trials without publications listed on ClinicalTrials.gov and all unregistered clinical trials classified as *Submitted*, *Fulfilled*, *Released*, or unclear (i.e., no status available) according to FDA or pharmaceutical company data, one reviewer (JDW) used a systematic two-step search strategy to locate publications (Google, Scopus (Elsevier), and PubMed) (June and July 2018), as has been done in prior research [7, 20]. A third reviewer (SSD) repeated all searches for postmarketing commitments that were determined to be unpublished.

### Statistical analysis

We used descriptive statistics to characterize the new drugs and biologics and postmarketing commitments. Analyses were performed using R (version 3.2.3; The R Project for Statistical Computing).

## Results

### Characteristics of new drugs and biologics and their postmarketing studies

Between 2009 and 2012, FDA approved 110 new drugs and biologics for 120 indications. While 97 (88.2%) had at least one postmarketing requirement at the time of first approval, 61 (55.5%) had at least one postmarketing commitment (Fig. 1); 5 (4.5%) had at least one postmarketing commitment and no postmarketing requirements. Among the 61 drugs and biologics for 68 total indications in the final sample (Table 1), 39 (63.9%) were drugs, 22 (36.1%) biologics, 19 (31.2%) were indicated for the treatment of cancer or hematologic disease, and 21 (34.4%) received priority review. There were 7 (11.5%) drugs and biologics that received accelerated approval and 14 (23.0%) that were designated as orphan products.

**Table 1:** Characteristics of 61 new drugs and biologics approved by the US Food and Drug Administration from 2009 through 2012 with at least one postmarketing commitment

Characteristic	No. (%)		
	Drugs *N* = 39	Biologics *N* = 22	Total *N* = 61
Approval year			
2009	9 (23.1)	6 (27.3)	15 (24.6)
2010	8 (20.5)	4 (18.2)	12 (19.7)
2011	12 (30.8)	6 (27.3)	18 (29.5)
2012	10 (25.6)	6 (27.3)	16 (26.2)
Therapeutic area			
Cancer and hematology	12 (30.8)	7 (31.8)	19 (31.2)
Infectious disease	6 (15.4)	0 (0.0)	6 (9.8)
Cardiovascular, diabetes, and hyperlipidemia	2 (5.1)	0 (0.0)	2 (3.3)
Autoimmune, musculoskeletal, and dermatology	2 (5.1)	9 (40.9)	11 (18.0)
Neurology and psychiatry	7 (17.9)	0 (0.0)	7 (11.5)
Respiratory	2 (5.1)	0 (0.0)	2 (3.3)
Gastrointestinal	2 (5.1)	0 (0.0)	2 (3.3)
Rare/metabolic diseases	3 (7.7)	1 (4.5)	4 (6.6)
Other^a^	3 (7.7)	5 (22.7)	8 (13.2)
Priority review			
Yes	21 (53.8)	0 (0.0)	21 (34.4)
No	18 (46.2)	22 (100.0)	40 (65.6)
Accelerated approval			
Yes	5 (12.8)	2 (9.1)	7 (11.5)
No	34 (87.2)	20 (90.9)	54 (88.5)
Orphan Drug Designation			
Yes	10 (25.6)	4 (18.2)	14 (23.0)
No	29 (74.4)	18 (81.8)	47 (77.0)

^a^“Other” includes contraception, hyponatremia, ophthalmology, and thalassemia syndromes

### Characteristics of postmarketing commitments

The FDA approval letters for these 61 drugs and biologics described 331 separate postmarketing commitments. The median number of commitments per approval letter was 3 (interquartile range [IQR], 1 to 7). While the total number of postmarketing commitments outlined in 2009, 2010, 2011, and 2012 were 65, 73, 89, and 104, respectively, the median number of commitments per approval letter for each new drug or biologic was 1, 1, 2, and 0, respectively. The majority of the postmarketing commitments (271 of 331 (81.9%)) were “Other studies,” including chemistry, manufacturing, and control (CMC) studies, which are related to the manufacturing and quality of products, including how products perform under different environmental conditions (Additional file 1: Box S3). Of the 49 (14.8%) commitments outlining a clinical trial, 33 described new clinical trials and 16 called for the submission of final reports or data from ongoing trials. Just over one quarter (89 (26.9%)) of the postmarketing commitments were subject to 506B reporting requirements, including 42 of 49 clinical trials (Table 2).

**Table 2:** Categories of postmarketing commitments for novel drugs and biologics approved by the US Food and Drug Administration between 2009 and 2012

Postmarketing commitment description	No. (%)		
	Subject to reporting requirements under 506B^a^		Total
	Yes	No^b^	
New clinical trials	27 (30.3)	6 (2.5)	33 (10.0)
Complete or submit results from ongoing clinical trials	15 (16.9)	1 (0.4)	16 (4.8)
Observational studies and secondary analyses^c^	11 (12.4)	0 (0.0)	11 (3.3)
Other studies^d^	36 (40.4)	235 (97.1)	271 (81.9)
Total	89 (26.9)	242 (73.1)	331

^a^Under section 506B of the Food and Drug Administration Modernization Act of 1997, FDA has the authority to monitor the progress of postmarketing studies concerning clinical safety, clinical efficacy, clinical pharmacology, or nonclinical toxicology, that applicants have agreed or are required to conduct

^b^Annual status reports to FDA could still be required under 21 CFR 314.81(b)(2)(viii) (drugs)

^c^Longer follow-up or new analyses of data from existing trials or studies; submission of a final report for ongoing case-control, cross-sectional, or retrospective cohort studies

^d^Manufacturing, stability, and immunogenicity studies without a primary safety endpoint; pharmacoepidemiologic studies; pharmacokinetic and/or pharmacodynamics trials; and chemistry, manufacturing, and controls study commitments that pharmaceutical companies have agreed with the FDA to conduct

Among the 33 postmarketing commitments for new clinical trials, the median number of words used to describe the study in publicly available documents was 42 (IQR, 29 to 63), thus providing limited information. Using only FDA approval letters, there was not enough information to establish use of randomization for 13 (39.4%), allocation for 20 (60.6%), comparator type for 27 (81.8%), outcome for 30 (90.9%), and number of patients to be enrolled for 32 (97.0%) of the 33 new clinical trials (Additional file 1: Table S1). In contrast, all 16 postmarketing commitments calling for the submission of final reports or data for ongoing trials included either a trial name or identifier.

Among the 27 postmarketing commitments for new clinical trials subject to 506B reporting requirements, 8 (29.7%) were classified as *Fulfilled* and 1 (3.7%) as *Released* using FDA’s Postmarketing Study and Clinical Trial Requirements and Commitments Database Files. According to the most recent database file, there were 4 (14.8%), 1 (3.7%), and 1 (3.7%) that were classified as *Delayed*, *Ongoing*, and *Submitted*, respectively (Additional file 1: Table S2**)**. There were 12 (44.4%) where we were unable to determine a recent (i.e., up-to-date) or closed (*Fulfilled* or *Released*) status in the archived Postmarketing Study and Clinical Requirements and Commitments Database Files. When all FDA supplemental letters and pharmaceutical company data were considered in addition to the databases, 11 (40.7%) were classified as *Fulfilled*, 4 (18.8%) as *Delayed*, 1 (3.7%) as *Ongoing*, 1 (3.7%) as *Released*, and 1 (3.7%) as *Submitted*. The remaining 9 (33.3%) did not have an up-to-date status. Publicly available pharmaceutical company data outlining a status were available for 9 (33.3%) 506B clinical trial postmarketing commitments (Additional file 1: Tables S2 and S3).

### Registration and study characteristics of clinical trials

Among the 33 postmarketing commitments for new clinical trials, two did not have enough information in their postmarketing commitment descriptions to perform ClinicalTrials.gov searches. Of the 31 remaining commitments, 28 (90.3%) were registered on ClinicalTrials.gov (Table 3). The majority of the 28 registered clinical trials were randomized (26, 92.9%) with double or triple blinding (22, 78.6%) (Table 4). Eighteen (63.0%) trials were placebo controlled and 4 (14.3%) had an active comparator. The majority of trials included efficacy primary endpoints that were categorized as clinical scales (17, 60.7%) and clinical outcomes (5, 17.9%); 6 (21.4%) focused on surrogate markers of disease. The median study duration and estimated or actual sample size according to the ClinicalTrials.gov registrations were 1.6 months (IQR, 0.9 to 12.0) and 400 patients (IQR, 254 to 529) respectively.

**Table 3:** Registration, results reporting, and publication of postmarketing commitments of new drugs and biologics approved by the Food and Drug Administration between 2009 and 2012

Category	No. (%)						
	Registration		Results reporting		Results reporting or peer-reviewed publication		
	Eligible for registration at ClinicalTrials.gov	Registered	Eligible for results reporting^a^	Results reported	Eligible for publication^b^	Published	Results reported or published
New clinical trials	31	28 (90.3)	23	22 (95.7)	29	14 (48.3)	22 (75.9)
Complete or submit results from ongoing clinical trials	16	16 (100.0)	15	15 (100.0)	15	14 (93.3)	15 (100.0)
Total	47	44 (93.6)	38	36 (94.7)	44	28 (63.6)	37 (84.1)

^a^Clinical studies classified as *Completed* or *Terminated* by ClinicalTrials.gov. The final rule requires the submission of results information no later than 1 year after the primary completion date

^b^We searched for publications for clinical trials classified by ClinicalTrials.gov as *Completed* or *Terminated* and by the FDA as *Submitted*, *Fulfilled*, *Released*, or unclear (e.g., last available record: 2013, ongoing)

**Table 4:** Study characteristics of 42 clinical trials registered on ClinicalTrials.gov based on ClinicalTrials.gov data

Characteristics	No. (%) or median (IQR)	
	New clinical trial *N* = 28	Complete or submit results from ongoing clinical trials *N* = 16
Randomization		
Yes	26 (92.9)	10 (62.5)
No	2 (7.1)	6 (37.5)
Allocation		
Double blind	22 (78.6)	6 (37.6)
Open label	6 (21.4%)	10 (62.5)
Comparator		
Placebo	18 (64.3)	6 (37.5)
Active	4 (14.3)	4 (25.0)
None	6 (21.4)	6 (37.5)
Endpoint		
Surrogate outcome	6 (21.4)	10 (62.5)
Clinical outcome	5 (17.9)	3 (18.8)
Clinical scale	17 (60.7)	0 (0.0)
Safety	0 (0.0)	3 (18.8)
Estimated sample size	400 (254 to 529)	237 (373 to 744)
Duration (months)	1.6 (0.9 to 12.0)	5.6 (5.2 to 12.6)

All 16 postmarketing commitments outlining the completion or submission of results from clinical trials were registered on ClinicalTrials.gov (Table 3). Of these, 10 (62.5%) were randomized, 6 (37.5%) were double or triple blind, 6 (37.5%) were placebo controlled, and 4 (25.0%) had an active comparator (Table 4). The majority of the trials (10, 62.5%) focused on surrogate outcomes. According to the ClinicalTrials.gov registrations, median study duration and estimated sample size were 5.6 months (IQR, 5.2 to 12.6) and 237 patients (IQR, 373 to 744), respectively.

### Results reporting and publication of clinical trials

Of the 23 postmarketing commitments for new trials classified as *Completed* or *Terminated* according to ClinicalTrials.gov, 22 (95.7%) had reported results (Table 3). Among the 29 registered or unregistered studies for which publication would be expected based on the most recent status provided by the FDA, pharmaceutical companies, or on ClinicalTrials.gov, just under half were published in a peer-reviewed journal (14 of 29 (48.3%)). Approximately three quarters (22 of 29 (75.9%)) had either reported results or were published. The median time from FDA approval to reported results or publication of new trials was 65 months (IQR, 47 to 81).

Among the 22 trials with reported results or a publication with a “report submission” date provided in the FDA approval letters, 18 (81.8%) reported results on ClinicalTrials.gov after the FDA scheduled submission deadline (median 13 (IQR, 4 to 24) months afterwards). There were 4 (18.9%) that reported results ahead of schedule (median 13 months (IQR, 10 to 16)). All 15 (100%) postmarketing commitments outlining the completion or submission of results from an ongoing study reported results, and all but one were published (14, 93.3%).

## Discussion

Just over half of the new drugs and biologics approved by the FDA between 2009 and 2012 had at least one postmarketing commitment outlined at the time of approval. These studies, which pharmaceutical companies agree to conduct, are not required by a statute or regulations, but may be a potentially important source of information about drug safety and effectiveness after market approval. However, the vast majority were not human subject research intended to gather additional information about the safety, efficacy, or optimal use of drugs and biologics in patients and were instead focused on product quality control, an important component of safety. Only 15% were clinical studies, less than one in ten new clinical trials. While these trials were nearly always registered with reported results on ClinicalTrials.gov, approximately one half had not yet been published in peer-reviewed journals and the vast majority reported results on ClinicalTrials.gov after FDA scheduled submission deadlines. Postmarketing commitments may offer an opportunity for FDA to work with pharmaceutical companies to generate important information about recently approved drugs and biologics after market approval.

Prior to FDAAA in 2007, when the term “postmarketing commitment” referred to all required, agreed-upon, and voluntary studies conducted by pharmaceutical companies after FDA drug approval, 74% of commitments were classified as clinical studies [5]. However, since 2008, FDA has distinguished between legally required studies and clinical trials (postmarketing requirements) and studies that “would not meet the statutory purposes” for postmarketing requirements (postmarketing commitments) [3]. Therefore, the low proportion of clinical trials identified as postmarketing commitments in our sample may not be surprising, considering that confirmatory, safety, and pediatric clinical trials can be formally required by FDA under the accelerated approval, FDAAA, and PREA postmarketing requirement authorities [7, 11], which were enacted in 1992, 2008, and 2003, respectively. According to a previous evaluation, approximately one quarter of postmarketing requirements for new drugs and biologics approved between 2009 and 2012 were for new clinical trials [7]. During this same time period, we found that 15% of postmarketing commitments were for clinical trials. Overall, the characteristics of the drugs and biologics with postmarketing commitments are similar to those previously observed among drugs and biologics with postmarketing requirements [7]. This likely reflects the fact that nearly one half of the new drugs and biologics approved between 2009 and 2012 had at least one postmarketing requirement and commitment. Furthermore, less than 5% had at least one postmarketing commitment and no requirements. Additional research is necessary to fully compare the characteristics of drugs and biologics with and without requirements or commitments and to establish the evidence gaps filled by postmarketing commitment studies.

Using the most recent FDA’s Postmarketing Study and Clinical Trial Requirements Database File and a limited number of archived databases (2009, 2010, 2011, 2016, 2017), we were unable to identify a recent (i.e., up-to-date) or closed (i.e., *Fulfilled* or *Released*) status for just under half of the postmarketing commitments for clinical trials subject to FDA reporting requirements under 506B. Pharmaceutical companies are required to provide the FDA with annual status reports for 506B studies of certain agreed-upon commitments, and FDA must publish annually in the Federal Register a report on the status of these postmarketing study commitments. While the Federal Register provides summary-level data, the FDA Postmarketing Study and Clinical Trial Requirements Database File outlines the individual postmarketing commitment or requirement statuses. These findings are consistent with a previous study suggesting that postmarketing requirements often lack a publicly available up-to-date status [7]. We also found that the rates of registration (90.3%) and results reporting (95.7%) on ClinicalTrials.gov among postmarketing commitment clinical trials were promising, which was similar to what has been previously observed among postmarketing requirements [7]. However, less than 50% of the completed clinical trials were published in a peer-reviewed journal, which suggests that the results from postmarketing commitment clinical trials may not be widely disseminated to clinical audiences, who often rely on the evidence reported in peer-reviewed publications. The low publication rate further highlights the importance of ClinicalTrials.gov as a potential source of clinical trial evidence.

Over 80% of the postmarketing commitments for new clinical trials reported results on ClinicalTrials.gov after FDA scheduled submission deadlines, which is higher than what has been previously observed among postmarketing requirements (68.1%) [7]. While FDA and pharmaceutical companies can revise the milestones outlined in the initial approval letters, these findings indicate that there are delays in study conduct, completion, and reporting, which may lead to gaps in the understanding of drug and biologic safety and effectiveness.

Prior studies have focused exclusively on the purposes and transparency of postmarketing requirements [7, 21–24]. Although postmarketing commitments are primarily for non-clinical studies, our work suggests that some commitments also generate clinical evidence. In order to further support FDA’s lifecycle evaluation process, FDA and pharmaceutical companies should continue to work closely to identify new clinical studies or other studies already being conducted, beyond the confirmatory and safety postmarketing requirement that can be required by FDA. However, greater transparency will be necessary to ensure that data from postmarketing commitments are able to inform care. For instance, longer and more detailed study descriptions will allow for the identification of specific study design characteristics, including endpoints, which are necessary to inform clinical practice, as well as more detailed explanations about the potential long-term knowledge gaps that are addressed. Furthermore, FDA could consider expanding its recent plans to add ClinicalTrials.gov identifiers to materials for future drug approvals [25, 26] to include postmarketing commitments, especially for those describing ongoing studies with trial identifiers. Similarly, ClinicalTrials.gov could include a variable specifying whether certain trials are postmarketing requirements or commitments. This will allow patients, clinicians, and researchers to locate and identify potential postmarketing studies and their results. In order to ensure that postmarketing commitment statuses are publicly identifiable, the FDA should keep all *Fulfilled* and *Released* 506B commitments on the Postmarketing Study and Clinical Trial Requirements Database, instead of removing them after 1 year of fulfillment or completion. Although postmarketing commitment trials were often registered with reported results, pharmaceutical companies can play a key role in promoting the dissemination of postmarket evidence by ensuring that the results of all agreed-upon clinical trials are published in peer-reviewed journals. Lastly, given the low publication rates, the FDA should make medical and/or summary reviews of postmarketing commitments and requirements publicly available, particularly for clinical trials. The FDA already releases this information for the clinical trials that are used to support the first approval of a new drug or biologic (i.e., pivotal trials).

### Limitations of this study

This study has some limitations. First, by limiting our study to new approvals between 2009 and 2012, we did not identify and classify all postmarketing commitments issued after FDAAA. By focusing on this time period, we were able to follow postmarketing commitments for at least 6 years for completion, result reporting, and publication of postmarketing commitments. However, it is possible that certain studies with durations longer than 6 years may not have had results reported or corresponding publications by the final follow-up of our study. In our sample, the median study duration among clinical trials was 1.6 months, and only one quarter of clinical trials had durations longer than 12 months. Therefore, we are reassured that our study allowed for an adequate amount of follow-up time for studies to be completed, reported, and published. However, it is possible that the unpublished studies had longer durations. Second, as previously discussed, our study was designed to rely on publicly available data sources, which made determining study design characteristics, ClinicalTrials.gov registrations, and corresponding publications difficult in some cases [7]. Although we attempted to be comprehensive by using numerous public data sources, we were unable to locate an up-to-date or closed status for nearly half of the commitments. While it is possible that a number of commitments classified as “unclear” are actually *Fulfilled* and *Released* commitments, which are only displayed on the FDA’s online database for 1 year after the date of fulfillment or release, our findings are a reflection of what information is made publicly available. Lastly, we used the milestone dates (e.g., postmarketing commitment protocol submission and study completion dates) outlined in the initial FDA approval letters. However, these dates can be changed, and pharmaceutical companies can submit revised schedules. The statuses provided in FDA’s Postmarketing Study and Clinical Trial Requirements Data Files do not always reflect the revisions and may have been established using the original study schedule [27]. Overall, the absence of an up-to-date status or milestone date could reflect a failure to report to the FDA by pharmaceutical companies, or FDA’s failure to review or update information in a timely manner.

## Conclusions

Among 331 postmarketing commitments outlined in approval letters for new drugs and biologics, the vast majority were for chemistry, manufacturing, controls, and other non-clinical studies. Only 15% of postmarketing commitments were new or ongoing clinical trials. While nearly half of postmarketing commitments subject to mandatory reporting requirements under Section 506B did not have a clear up-to-date progress reported publicly in FDA sources, the majority of clinical trials were registered on ClinicalTrials.gov with reported results. However, only half of the clinical trials had corresponding publications in peer-reviewed journals. Opportunities may exist for FDA and pharmaceutical companies to work together to identify additional postmarketing commitments that support FDA’s lifecycle evaluation process by generating information about the safety, efficacy, or optimal use of drugs and biologics in patients. Additional postmarketing commitments for clinical trials can help generate data that ensures that patients and physicians have better information to guide clinical decisions, beyond addressing issues typically examined by postmarketing requirements.

## Additional file

Additional file 1: Box S1. (The history of US Food and Drug Administration’s postmarketing commitments and requirements). Box S2. (Postmarketing commitment reporting requirements). Box S3. (Postmarking commitment categorization). Box S4. (FDA postmarketing commitment status categories). **Table S1.** (Postmarketing commitment status based only on FDA’s Postmarket and Commitment Database for new drugs and biologics subject to 506B reporting requirements). **Table S2.** (Study characteristics of 33 new clinical trials based on postmarketing commitment descriptions in FDA approval). **Table S3.** (Postmarketing commitment status based on FDA’s Postmarket and Commitment Database, supplementary approvals, and sponsor materials for new drugs and biologics subject to 506B reporting requirements). (PDF 181 kb)

## Abbreviations

CI: Confidence interval
FDA: Food and Drug Administration
IQR: Interquartile range
