# Supplementary material for: Postmarketing commitments for novel drugs and biologics approved by the US Food and Drug Administration: a cross-sectional analysis
Source: BMC Med. 2019 Jun 17;17:117. doi: 10.1186/s12916-019-1344-3 (PMC6572730; doi:10.1186/s12916-019-1344-3)
Supplement: Supplementary file 1 — Box S1. (The history of US Food and Drug Administration’s postmarketing commitments and requirements). Box S2. (Postmarketing commitment reporting requirements). Box S3. (Postmarking commitment categorization). Box S4. (FDA postmarketing commitment status categories). Table S1. (Postmarketing commitment status based only on FDA’s Postmarket and Commitment Database for new drugs and biologics subject to 506B reporting requirements). Table S2. (Study characteristics of 33 new clinical trials based on postmarketing commitment descriptions in FDA approval). Table S3. (Postmarketing commitment status based on FDA’s Postmarket and Commitment Database, supplementary approvals, and sponsor materials for new drugs and biologics subject to 506B reporting requirements). (PDF 181 kb) [file 12916_2019_1344_MOESM1_ESM.pdf]

SUPPLEMENTARY TABLES AND BOXES

Postmarketing commitments for new drugs and biologics approved by the US Food and Drug Administration: a cross-sectional analysis

Joshua D Wallach; Anita T Luxkaranayagam; Sanket S Dhruva; Jennifer E Miller; Joseph S Ross

Contact: Joshua.wallach@yale.edu

**SUPPLEMENTARY APPENDIX FILE**	**Page #**
**SUPPLEMENTARY BOX 1**: The history of US Food and Drug Administration's postmarketing commitments and requirements	**2**
**SUPPLEMENTARY BOX 2:** Postmarketing commitment reporting requirements	**3**
**SUPPLEMENTARY BOX 3:** Postmarking commitment categorization	**4**
**SUPPLEMENTARY BOX 4**: FDA postmarketing commitment status categories	**5**
**SUPPLEMENTARY TABLE 1.** Postmarketing commitment status based only on FDA's Postmarket and Commitment Database for new drugs and biologics subject to 506B reporting requirements	**6**
**SUPPLEMENTARY TABLE 2.** Study characteristics of 33 new clinical trials based on postmarketing commitment descriptions in FDA approval letters	**7**
**SUPPLEMENTARY TABLE 3.** Postmarketing commitment status based on FDA's Postmarket and Commitment Database, supplementary approvals, and pharmaceutical company materials for new drugs and biologics subject to 506B reporting requirements	**8**
**Determinations based on previous studies**	**9**

**SUPPLEMENTARY BOX 1. The history of US Food and Drug Administration's postmarketing commitments and requirements**	
**Before FDAAA[a]**	**After FDAAA**
Required, agreed upon, and voluntary studies were classified as "*postmarketing commitments*"	Required studies were classified as "*postmarketing requirements*" and agreed upon and voluntary studies were classified as "*postmarketing commitments*"
**Statutory or regulatory authorities used to require postmarketing studies**	
- *Animal Efficacy Rule*: Novel drugs can be approved when human efficacy studies and field trials may not be ethical and/or feasible, but FDA may require postmarket studies in humans<br>- *Pediatric Research Equity Act*: FDA can approve novel drugs for use in adults without corresponding studies for the same indication in the relevant pediatric population, but FDA can include deferred pediatric studies or clinical trials as postmarketing requirements<br>- *Accelerated Approval:* To expedite the approval of novel drugs that treat serious diseases and that fill unmet medical needs on the basis of surrogate or intermediate endpoints "reasonably likely" to predict clinical benefit,[12] FDA has the authority to require postmarket studies to confirm efficacy	- *Animal Efficacy Rule*<br>- *Pediatric Research Equity Act*<br>- *Accelerated Approval*<br>- *Food and Drug Administration Amendments Act (FDAAA) Section 505(o)(3):* To provide additional information for novel therapeutics approved under section 505 of FDAAA or section 351 of the Public Health Services Act, FDA can require postmarket studies that assess know serious risks, signs of serious risks, or unexpected serious risked related to the use of a novel drug
FDAAA = Food and Drug Administration Amendments Act<br>[a] Section 901 of FDAAA went into effect on March 25, 2008	

**Supplementary Box 2. Postmarketing commitment reporting requirements**		
**506B (Reports of Postmarketing Studies)**	**Study design examples**	**Reporting requirements[13]**
Yes	- clinical safety<br>- clinical efficacy<br>- clinical pharmacology<br>- nonclinical toxicology	- Pharmaceutical companies are required to provide FDA with annual reports on the status of the 506B studies<br>- FDA must publish annually in the *Federal Register* a report on the status of postmarketing study commitments
No	- Chemistry, manufacturing, and controls (CMC)<br>- Product stability studies<br>- Voluntary studies	- BLAs: Non-506B studies are not subject to reporting requirements that are described under 21 CFR 601.70<br>- NDAs: Applicant is required to advise FDA on that status of non-506B studies in separate section of the NDA annual report (21 CFR 314.81(b)(2)(viii)). Voluntary commitments are not subject to 506B reporting requirements.[13]
BLA = Biologics License Application; NDA = New Drug Application<br>[a] Code of Federal Regulations (CFR), Title 21, Volume 7, Biologics, 21 CFR 601.70<br>[b] Code of Federal Regulations (CFR), Title 21, Volume 5, Drugs, 21 CFR 314.81(b)(2)(vii) and (viii)		

Supplementary Box 3. Postmarking commitment categorization	
Definition	***Example***
New Clinical Trials	
Postmarketing commitments that outline *new* clinical trials, including randomized and non-randomized clinical trials evaluating efficacy or "efficacy and safety". This includes "clinical trials in which the primary endpoint is related to further defining efficacy, designed to: evaluate long-term effectiveness or duration of response, evaluate efficacy using a withdrawal design, evaluate efficacy in a subgroup."[4]	"Randomized, double-blind, adequate and well controlled, multiple fixed-dose, parallel group clinical trial of Xeomin (incobotulinumtoxinA) in botulinum toxin-naïve children age 2-17 years with upper extremity spasticity. The minimum duration of the trial should be 12 weeks. You should propose a method to actively monitor for adverse events related to spread of toxin. The protocol for the trial should be submitted to the FDA as a special protocol assessment (SPA)."
Complete or submit results from ongoing clinical trials	
Instead of requesting *new* clinical trials, these postmarketing commitments call for the completion and submission of the results from ongoing clinical trials.	"To submit the final report for clinical trial Hx-CD20-406 entitled "A single-arm international, multi-center trial of HuMax-CD20, a fully human monoclonal anti-CD20 antibody, in patients with B-cell Chronic Lymphocytic Leukemia who have failed fludarabine and alemtuzumab" which shall include results of objective response rates according to the IRC and according to the clinical investigators. The final report will provide summary analyses and primary data. Accrual to this trial has been completed."
Observational studies, analyze/follow-up from clinical studies, and other flexible commitments	
Postmarketing commitments that outline longer follow-up or new analyses of data from existing trials or studies; submission of a final report for ongoing case-control, cross-sectional, or retrospective cohort studies.	"Exposure-Response Analyses Assessment: Submit an exposure-response analysis for regorafenib and its active metabolites M2 and M5 using data collected from the CORRECT trial (Study 14387) in patients with metastatic colorectal cancer (mCRC) who have progressed after standard therapy."
Other studies	
Manufacturing, stability, and immunogenicity studies that do not have a primary safety endpoint; pharmacoepidemiologic studies; pharmacokinetic and/or pharmacodynamics trials; and chemistry, manufacturing, and controls (CMC) study commitments that sponsors have agreed with the FDA to conduct (e.g., CMC commitments relate to the quality and manufacturing of products (i.e., stability testing to determine how the substance performs under different temperatures and light intensities).	"An in vitro study to assess the potential for carglumic acid to inhibit or induce cytochrome P450 enzymes."

**SUPPLEMENTARY BOX 4. FDA postmarketing commitment status categories**[1]	
**Open Status Categories**	
Pending	"The study/clinical trial has not been initiated (i.e., no subjects have been enrolled or animals dosed), but does not meet the criterion for *delayed* (i.e., the original projected date for initiation of patient accrual or initiation of animal dosing has not passed)."[1]
Ongoing	"The study/clinical trial is proceeding according to, or is ahead of, the original schedule. The FDA considers a study/clinical trial to be ongoing until a final report is submitted to the FDA, as long as the activities are proceeding according to the original schedule. If patient accrual or animal dosing has started but is not complete, and the projected date for completion of that milestone has passed, the study/clinical trial should be categorized as *delayed*."[1]
Delayed	"The progression of the study/clinical trial is behind the original schedule. Delays can occur in any phase of the study/clinical trial, including patient enrollment, analysis of results, or submission of the final report to the FDA. While the original schedule—not a revised schedule—serves as the basis for defining a study/clinical trial as delayed, each phase of the study/clinical trial will be considered in its own right. If the applicant has one delayed phase, but gets back on schedule during the next phase, the delayed status will no longer apply."[1]
Terminated	"The applicant ended the study/clinical trial before completion and has not yet submitted a final report to the FDA."[1]
Submitted	"The applicant has concluded or terminated the study/clinical trial and has submitted a final report to the FDA, but FDA has not yet notified the applicant in writing that the requirement/commitment has been fulfilled or that the requirement/commitment has been released."[1]
**Closed Status Categories**	
Fulfilled	"The applicant has submitted the final report for the requirement/commitment, and, upon review of the final report, FDA is satisfied that the applicant has met the terms of the requirement/commitment."[1]
Released	"FDA has informed the applicant that it has been released from its requirement/commitment to conduct the postmarketing study/clinical trial because it is either no longer feasible or would no longer provide useful information."[1]

**SUPPLEMENTARY TABLE 1. Study characteristics of 33 new clinical trials based on postmarketing commitment descriptions in FDA approval letters**	
**Characteristics**	No. (%)
**Randomization**	
Randomized	19 (57.6)
Non-randomized	1 (1.0)
Unclear	13 (39.4)
**Allocation**	
Double blind	12 (36.4)
Single blind	0 (0.0)
Open label	1 (1.0)
Unclear	20 (60.6)
**Comparator**	
Placebo	4 (12.1)
Active	1 (3.0)
None	1 (3.0)
Unclear	27 (81.8)
**Outcome**	
At least one	3 (9.1)
None	30 (90.9)
**Duration**	
Exact or approximate	1 (3.0)
Minimum[a]	10 (30.3)
Unclear	22 (66.7)
**Number of patients to be enrolled**	
Exact or approximate	0 (0.0)
Minimum[a]	1 (3.0)
Unclear	32 (97.0)
[a] This includes postmarketing commitment descriptions that outline a total minimum enrollment or a minimum enrollment for the treatment arm only.	

**SUPPLEMENTARY TABLE 2. Postmarketing commitment status based only on FDA's Postmarket and Commitment Database for new drugs and biologics subject to 506B reporting requirements**									
	**Numbers based on current data, number based on old data[a]**								
**Postmarketing Commitments**	**Status[b]**								**Total**
All (N = 89)	Pending	Ongoing	Delayed	Terminated	Submitted	Fulfilled	Released	Unclear	
New clinical studies (N =27)	0, 1	1, 2	4, 0	0	1, 2	8	1	7	27
Complete or submit results from ongoing clinical studies (N =15)	0, 2	0, 3	0, 1	0	1, 1	3	0	4	15
Observational studies and secondary analyses (N =11)	1, 1	0, 0	0, 0	0	0, 0	2	0	7	11
Other studies (N =36)	0, 8	0, 1	0, 1	0	0,1	7	1	17	
Total separated	1, 12	1, 6	4, 2	0	2, 4	20	2	35	89
Total combined	13	7	6	0	6	20	2	35	89

[a] The second value represents the last available status for postmarketing commitments without a clear up-to-date status. Archived FDA Postmarketing Study and Clinical Trial Requirements and Commitments Database Files were used to determine the most recent status and date for each postmarketing commitment (e.g., "last available status: *Pending,* October 31, 2010).

Among the 89 postmarketing commitments subject to 506B reporting requirements, 20 (22.5%) were classified as fulfilled according to FDA Postmarketing Study and Clinical Trial Requirements Database files. Over two-thirds (59 of 89 (66.3%)) of the 506B studies did not have enough information in the databases to determine an up to data status; 35 of these had no past or current status (**Additional file 1: Supplementary table 2**).

**SUPPLEMENTARY TABLE 3. Postmarketing commitment status based on FDA's Postmarket and Commitment Database, supplementary approvals, and pharmaceutical company materials for new drugs and biologics subject to 506B reporting requirements**											
			**Numbers based on current data, number based on old data[a]**								
**Postmarketing Commitments**			**Status[b]**								**Total**
All (N = 89)	Number with pharmaceutical company status[c]	Number with status in supplemental letters[b]	Pending	Ongoing	Delayed	Terminated	Submitted	Fulfilled	Released	Unclear	
New clinical studies (N =27)	9	6	0, 2	0, 2	4, 0	0, 0	1, 0	11	2	5	18, 9
Complete or submit results from ongoing clinical studies (N =15)	6	4	0, 1	0, 2	0, 0	0, 0	1, 0	9	0	2	10, 5
Analyze/follow-up from clinical studies (and other flexible commitments) (N =11)	4	4	0, 1	1, 0	0, 0	0, 0	0, 0	6	0	3	7, 4
Other studies (N =36)	9	6	0, 5	0, 1	0, 0	0, 0	0, 1	14	2	13	16, 20
Total separated	28	20	0, 9	1, 5	4, 0	0, 0	2, 1	40	4	23	51, 38
Total combined			9	6	4	0	3	40	4	23	89

[a] The second value represents the last available status for postmarketing commitments without a clear up-to-date status. Archived FDA Postmarketing Study and Clinical Trial Requirements and Commitments Database Files were used to determine the most recent status and date for each postmarketing commitment (e.g., "last available status: *Pending,* October 31, 2010).
[b] 19 postmarketing commitments were classified as fulfilled according to supplemental letters on Drugs@FDA and 9 of 19 did not have a status provided by the FDA Postmarketing Study and Clinical Trial Requirements and Commitments Database Files.
[c] 28 postmarketing commitments had a status provided by the pharmaceutical company and 3 of 28 did not have a status provided by the FDA Postmarketing Study and Clinical Trial Requirements and Commitments Database Files nor FDA supplemental letters.
25 postmarketing commitments had status information provided by both FDA and pharmaceutical company data. The FDA and pharmaceutical company data provided different statuses for 15 of 25 postmarketing commitments. Of these, 5 were described as further along according to pharmaceutical company data.

Determinations based on previous evaluations[5, 6]

Determining trial duration:

1. When available, duration was based on the length of time in weeks between the time when the first dose of the drug was administered and the time that the primary endpoint was measured. Two examples are used to illustrate how trial length is calculated
    a. Trial lasting one year in which the primary outcome is measured at 24 weeks: trial length = 24 weeks
2. If not 1, when available, we determined the median follow-up for time to event endpoints. When differences in trial length existed between treatment arms, a weighted average calculation was used to determine a single value for trial length.
3. If not 2, we recorded the final cut-off time.

Comparator:

1. **No comparator:** Single arm trials and multi-arm trials comparing the drug to itself only
2. **Placebo:** Trials comparing he study drug to a placebo only
3. **Active:** Trials comparing the study drug to another drug. Trials comparing the study drug to both an active comparator and placebo were included in the classification.

**FDAAA applicable trials**
"Applicable clinical trials" are those subject to mandatory registration and reporting requirements under the Food and Drug Administration Amendments Act (FDAAA). Generally, this includes 'controlled clinical investigation(s), other than a phase I clinical investigation, of a drug subject to section 505 of the Federal Food, Drug, and Cosmetic Act or to section 351 of this Act'.[2] Furthermore, these trials should have been 'either initiated after 27 September 2007, or initiated on or before that date and were still ongoing as of 26 December 2007',[2] and meet one of the following conditions:<br>A. The trial has one or more sites in the USA,<br>B. The trial is conducted under an FDA investigational new drug application (IND), or<br>C. The trial involves a drug or biological that is manufactured in the USA or its territories and is exported for research.[2-4]<br><br>As outlined on ClinicalTrials.gov, according to the Final Rule for Section 801 of FDAAA, "noninterventional (observational) clinical research (such as cohort or case-control studies)" are generally excluded from the registration and results submission requirements.[3]

REFERENCES

1. U.S. Food and Drug Administration. Postmarketing Requirements and Commitments **(FAQ).** https://www.fda.gov/Drugs/GuidanceComplianceRegulatoryInformation/Post-marketingPhaseIVCommitments/ucm070766.htm**. Accessed 10 Oct 2018.** In.

2. Food and Drug Administration Amendments Act of 2007. **US Public Law 110-85. (2007, Sept 27); 21 USC 301**. In.

3. ClinicalTrials.gov**. FDAAA 801 requirements.** http://clinicaltrials.gov/ct2/manage-recs/fdaaa - /WhichTrialsMustBeRegistered. In.

4. Miller JE, Korn D, Ross JS: **Clinical trial registration, reporting, publication and FDAAA compliance: a cross-sectional analysis and ranking of new drugs approved by the FDA in 2012**. *BMJ Open* 2015, **5**(11):e009758.

5. Downing NS, Aminawung JA, Shah ND, Krumholz HM, Ross JS: **Clinical trial evidence supporting FDA approval of novel therapeutic agents, 2005-2012**. *JAMA* 2014, **311**(4):368-377.

6. Wallach JD, Egilman AC, Dhruva SS, McCarthy ME, Miller JE, Woloshin S, Schwartz LM, Ross JS: **Postmarket studies required by the US Food and Drug Administration for new drugs and biologics approved between 2009 and 2012: cross sectional analysis**. *BMJ* 2018, **361**:k2031.
